# Downregulation of Sirt1 is correlated to upregulation of p53 and increased apoptosis in epicardial adipose tissue of patients with coronary artery disease

**DOI:** 10.17179/excli2020-2423

**Published:** 2020-10-02

**Authors:** Mahdieh Khanahmadi, Babak Manafi, Heidar Tayebinia, Jamshid Karimi, Iraj Khodadadi

**Affiliations:** 1 Department of Clinical Biochemistry, Faculty of Medicine, Hamadan University of Medical Sciences, Hamadan, Iran; 2 Department of Surgery, Faculty of Medicine, Hamadan University of Medical Sciences, Hamadan, Iran

**Keywords:** apoptosis, atherosclerosis, coronary artery disease, epicardial adipose tissue, Sirt1, p53

## Abstract

The higher expression level of p53 in epithelial adipose tissue (EAT) has previously been reported in atherosclerosis. Since we hypothesized that the expression of p53 is modulated by Sirt1, the aim of this study was to determine the expression levels of Sirt1 and p53 and to investigate their correlation to apoptosis in EAT of patients with coronary artery disease (CAD). Thirty-five patients with more than 50 % stenosis in at least one of the main coronary arteries were considered as CAD group while 29 patients with no clinical signs of atherosclerosis who underwent open-heart surgery for valve replacement were classified as control group. EAT biopsy samples were collected from all participants during surgery. Sirt1, p53, Bax, and Bcl-2 gene expression levels were determined in EAT by qRT-PCR and Western blotting was carried out to assess Sirt1 and p53 protein levels. Hematoxylin and eosin staining was used for histopathological analysis. mRNA and protein levels of Sirt1 in EAT were significantly lower in patients with CAD compared with control group, whereas CAD patients showed greater p53 gene and protein expressions. In addition, inverse correlations were observed between Sirt1 and p53 at both mRNA and protein levels. The Bax and ratio of Bax/Bcl-2 gene expressions were higher in CAD group, but no difference was observed in Bcl-2 expression. Histopathological analysis showed apoptotic bodies and infiltrated immune cells in EAT of CAD group. Our results suggest that the Sirt1-p53 axis may involve in atherosclerosis by promotion of apoptosis.

## Introduction

Cardiovascular disease (CVD) is the main cause of 31 % of deaths worldwide. According to the World Health Organization (WHO) report, 17.9 million people die each year from CVD. The most common type of CVD is coronary artery disease (CAD), an inflammatory condition that entangles coronary vessels (Hansson and Hermansson, 2011[15]; Huang et al., 2019[17]). 

Atherosclerosis is a progressive chronic inflammatory process that leads to the deposition of cholesterol and lipid-loaded macrophages in artery walls and more often results in subsequent formation of atherosclerotic plaques (Abdolmaleki et al., 2019[1]; Li et al., 2019[21]). Studies have shown that macrophages have key roles in atherosclerosis from initiation phase to the developed stage (Gonzalez and Trigatti, 2017[12]). Phagocytizing of and cell debris and up-taking of lipoproteins, particularly oxidized-LDL by macrophages in the atherosclerotic plaque evokes acute thrombosis (Andres et al., 2012[3]; Tabas, 2009[33]).

Among factors affecting CAD, epicardial adipose tissue (EAT) which is in direct contact with the myocardium and its surrounding coroner vessels (Gaborit et al., 2017[11]), has a significant role in the pathogenesis of CAD due to its dynamic and secretory nature by producing a wide array of cytokines and adipokines (Iacobellis et al., 2005[19]; Iacobellis and Bianco, 2011[18]). Although these secretions have gradual effects on the development of atherosclerosis, the exact molecular mechanism of the involvement of EAT in atherosclerosis has been remained puzzling and unclear. 

Sirtuins (silent mating type information regulation 2 homolog) are members of the third subgroup of histone deacetylase family with Sirt1 as the most known and the most studied protein. Sirtuins exhibit deacetylase and mono-ribosyl transferase activities and involve in the regulation of lipid metabolism in skeletal muscle, liver, and adipose tissue (Feige and Auwerx, 2008[10]; Lomb et al., 2010[24]; Michan and Sinclair, 2007[26]). Contribution of Sirt1 in various metabolic pathways such as the involvement of Sirt1 in the activation of cellular tumor antigen p53 in EAT has previously been shown (Agra et al., 2014[2]; Haigis and Guarente, 2006[14]). Sirt1 deacetylates p53 and inactivates its function in promotion of apoptosis, and therefore suppresses the progression of atherosclerosis (Haigis and Guarente, 2006[14]). Interestingly, a higher level of active p53 has been reported in EAT in patients with heart failure (Agra et al., 2014[2]). Therefore, it is hypothesized that the higher activity of p53 in CAD patients might be due to the reduction in the expression of Sirt1. Because of contribution of p53 in apoptosis, it is also believed that the higher level of p53 (lower Sirt1) might be concurrent with the higher apoptosis in coronary artery disease. Thus, the present study for the first time, investigated the correlation between the expression of Sirt1 and p53 at both mRNA and protein levels and determined the expression of genes involved in apoptosis in epicardial adipose tissue of patients with coronary artery disease. 

## Materials and Methods

### Study population

A total of sixty-four patients undergoing open-heart surgery in Farshchian Cardiovascular Subspecialty Hospital (Hamadan-Iran) to complete the treatment process were enrolled in this case-control study. Thirty-five patients (23 men and 12 women) with clinical signs of atherosclerosis) having over 50 % stenosis in at least one of the main coronary arteries) were taken as volunteer subjects for coronary artery bypass grafting and were considered as CAD group (test group). All patients in CAD group were under treatment using appropriate medications. On the other hand, 29 age- and BMI-matched patients (13 men, 16 women) with normal coronary and no clinical signs of atherosclerosis (having less than 50 % stenosis) who underwent mitral valve replacement were assigned as control group. Clinical diagnosis for all subjects was confirmed by specialists during angiographic examinations. 

Patients with any sign of liver disease, renal failure, or neoplastic diseases and patients under steroid therapy were excluded from the experiment. Diabetes mellitus, recent acute myocardial infarction, and recent gastrointestinal surgery were also considered as exclusion criteria. Demographic characteristics and medications consumed by patients were recorded for each and every patient. 

The study was approved by Research Ethics Committee of Hamadan University of Medical Sciences (Hamadan-Iran). All procedures performed in the study were in accordance with the ethical guidelines of the Declaration of Helsinki (1967; version 2013) and with the ethical standards of the national Iranian research committee. The nature of the study was explained to patients and written consents were obtained from all applicants.

### Sample collection

In the morning of surgery, intravenous blood samples were collected, centrifuged at 1500⨯g for 20 min, and serum samples were separated and stored in aliquots at -80 °C for biochemical analysis. Epicardial adipose tissue samples were dissected from the proximal tract of the right coronary artery prior to surgery. In order to eliminate blood contamination, tissue samples were rinsed with phosphate-buffered saline (PBS) and divided into three portions. Two of which were frozen immediately in liquid nitrogen or stored at −80 °C for RNA extraction and Western blotting, respectively and the third portion was immersed in neutralized formalin for histological examination. 

### Analysis of biochemical parameters 

Fasting blood sugar and serum total cholesterol, triglyceride, LDL-C, and HDL-C were determined using Pars Azmoon commercial kits (Pars Azmoon, Tehran-Iran). 

### Quantitative RT-PCR for gene expression assay

Epicardial Sirt1, p53, Bax, and Bcl-2 gene expression levels were determined by qRT-PCR using RealQ Plus Master Mix Green (Ampliqon, Odense-Denmark) on a Roche LightCyclerVR 96 System (Roche Life Science Deutschland GmbH, Sandhofer-Germany). Briefly, total RNA was isolated using phenol-chloroform method and was quantified using NanoDrop^TM^ (Thermo Fisher Scientific, USA). The quality and integrity of total RNA were confirmed by 1 % agarose gel electrophoresis. cDNA was synthesized by reverse transcription of 500 ng of total RNA using HyperScriptTM Reverse Transcriptase cDNA Kit (Gene All Biotechnology Co. Ltd., Korea). Specific primers were designed using Allele ID^®^ software (Premier Biosoft Corporation, USA) as forward: 5′-TAGGCGGCTTGATGGTAATC-3′ and reverse: 5′-TGGCATGTCCCACTATCACT-3′ primers for Sirt1 while a set of forward: 5'-TAACAGTTCCTGCATGGGCGGC-3' and reverse: 5'-AGGACAGGCACAAACACGCACC-3' primer was used for p53 gene. Forward and reverse primer set for Bax was 5′-CCGCCGTGGACACAGACT-3′ and 5′-TTGAAGTTGCCGTCAGAAAACA-3′, respectively and for Bcl-2 a pair of forward: 5′-TGGAGAGTGCTGAAGATTGA-3′ and reverse: 5′GTCTACTTCCTCTGTGATGTTGTAT-3′ primers were used. For housekeeping gene, β-actin was used with forward and reverse primer sequences as 5'-ACAGAGCCTCGCCTTTGC-3' and 5'-ATCACGCCCTGGTGCCT-3'. Finally, the relative mRNA expression levels (fold change) of Sirt1, p53, Bax, and Bcl-2 were calculated as 2^−^^△^^Ct^ compared with the expression of β-actin. 

### Western blotting for protein assay

Total protein of Epicardial adipose tissue was extracted using a lysis buffer containing 600 μl of RIPA (Thermo Scientific, USA) and 6 μl of the protease inhibitor (Sigma Aldrich, US) with bovine serum albumin as reference protein. Briefly, 80 mg of frozen EAT samples were powdered with liquid nitrogen, the lysis buffer was added and homogenized. The homogenates were placed on a 4 °C shaker for 2 h and then centrifuged at 12000⨯g for 20 min. The supernatants containing cell soluble proteins were transferred to new microtubes and protein concentrations were measured using Bicinchoninic Acid (BCA) protein assay kit (Thermo Scientific, USA).

An equal amount (50 µg) of total protein from each sample was separated by electrophoresis on SDS-PAGE gels, then proteins were transferred from the gel to the UltraCruz® Nitrocellulose Pure Transfer Membrane. After blocking with TBST buffer (Tris buffered Saline-Tween 20 containing 5 % lean milk) for 2 h at room temperature, the membranes were incubated with primary antibodies against p53 (United Kingdom, St John's Laboratory, STJ94890), Sirt1 (United Kingdom, St John's Laboratory, STJ95667), and β-actin (Abcam, ab8227) for 12 h. Membranes were then washed three times with TBST buffer for 15 min and incubated with the secondary antibody (Abcam, USA, ab191866) for 1 h. Membranes were then stained with ECL (Bio-Rad, Germany) and the protein bands were exposed to the film in the darkness. Finally, the relative protein expression levels were quantified by ImageJ software version 1.46 (https:// imagej.nih.gov/ij/) and normalized to β-actin as control.

### Histopathological analysis of EAT

For histopathological evaluation, formalin-fixed EAT samples were embedded in paraffin, sectioned at 5 µm thicknesses and stained with hematoxylin-eosin for light microscopic examination.

### Statistical analysis

Data analysis was performed using SPSS software version 18.0 (SPSS Inc., Chicago, IL-USA) and Graph Pad Prism software version 6.0 (Graph Pad Software, CA-USA). Kolmogorov-Smirnov test, Mann Whitney U test, and Student's t-test were used as appropriate. Spearman's correlation coefficient (r) was used to examine the relationship between the variables. Data was expressed as [mean±SD] or [median (IQR)], as appropriate and p value less than 0.05 was considered as statistically significant difference. IQR represented interquartile range.

## Results

### Demographic characteristics and biochemical parameters

As shown in Table 1(Tab. 1), demographic characteristics revealed no significant differences in age, sex, BMI, and family history between CAD and control groups. There was also no significant difference in the frequency of smoking and exercise between two groups. As expected, hyperlipidemia and hypertension were more predominant in CAD patients than control subjects. Fasting blood sugar, total cholesterol, triglyceride, and LDL-C did not differ between groups but HDL-C was significantly lower in the CAD group than in the control group (p = 0.002). Patients in CAD group were under treatment using appropriate medications (Supplementary data). See also Supplementary material.

### Sirt1 regulates the expression of p53 in patients with coronary artery diseases

**Figure 1:**
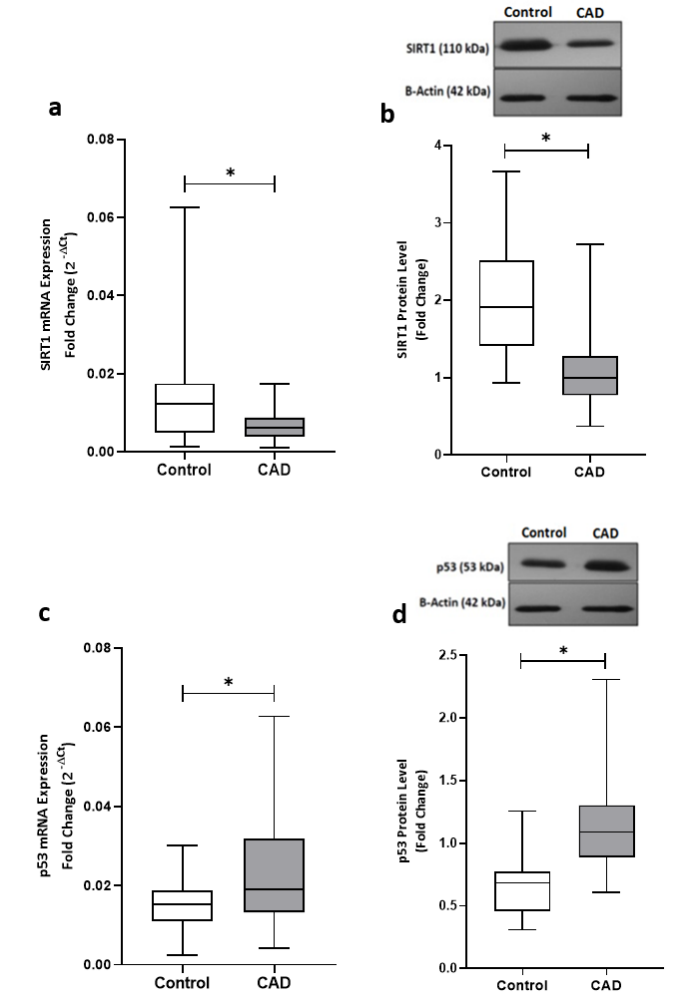
The gene expression and protein levels of Sirt1 and p53 in EAT of CAD patients and control group. The gene expression (a) and protein level (b) of Sirt1 were significantly lower in CAD patients (n=35) compared with the control group (n=29). P53 gene expression (c) and protein level (d) were found significantly higher in CAD patients. Gene expressions were determined by qRT-PCR whereas protein levels were measured by Western blotting and quantified densitometrically using ImageJ software. The results are reported as median (IQR). Asterisk (*) represents p<0.05 and IQR is interquartile range.

**Figure 2:**
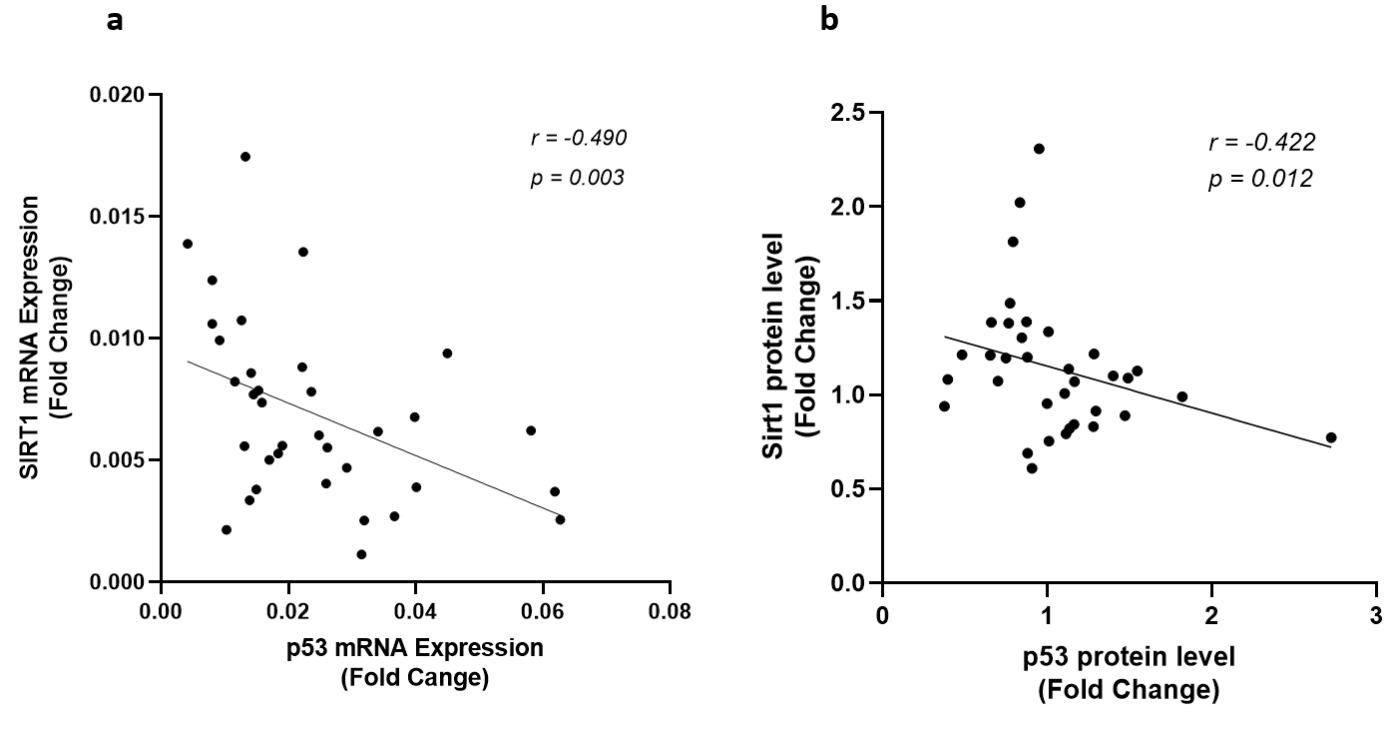
Correlation of Sirt1 and p53 expression levels. (a) Correlation of Sirt1 and p53 gene expression levels and (b) protein levels of Sirt1 and p53 in EAT of CAD patients (n=35) compared with the control group (n=29). Gene expressions were determined by qRT-PCR whereas protein levels were measured by Western blotting and quantified densitometrically using ImageJ software. Spearman's correlation coefficient (r) was used to examine the relationship between the variables.

Sirt1 expression at both mRNA and protein levels were nearly 50 % lower in epicardial adipose tissue of CAD patients compared to the patients from control group, as shown in Figure 1(Fig. 1). In contrast, the gene expression and the protein level of p53 were found significantly upregulated in patients with coronary artery diseases (Figure 1(Fig. 1)). Interestingly, linear regression analysis confirmed the presence of an inverse correlation between the Sirt1 and p53 at both the gene expression (r = - 0.490, p = 0.003) and protein (r = - 0.422, p = 0.012) levels and greater levels of Sirt1 were found correlated to the lower levels of p53, as shown in Figure 2(Fig. 2).

### The expression of the genes involved in apoptosis in CAD

**Figure 3:**
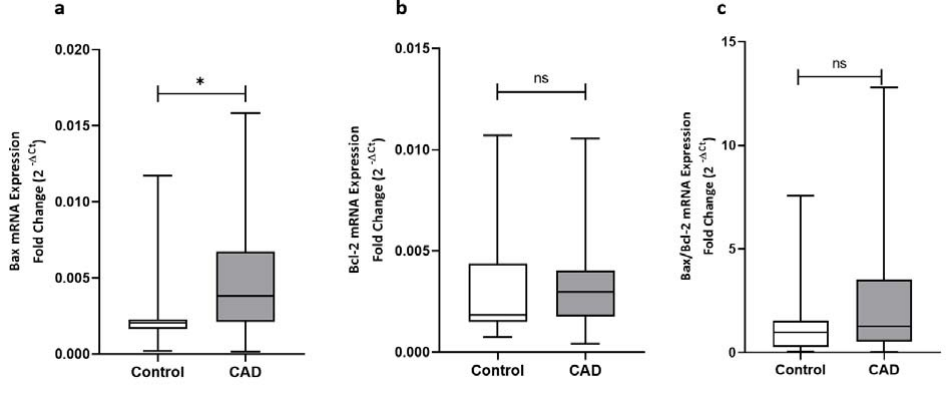
The gene expression levels of Bax, Bcl-2, and the Bax/Bcl-2 ratio of gene expression. The gene expression levels of Bax (a), Bcl-2 (b), and the Bax/Bcl-2 ratio of gene expression (c), as determined by qRT-PCR in EAT of CAD patients (n=35) compared with the control group (n=29). The results are reported as median (IQR). Asterisk (*) represents p<0.05 and IQR is interquartile range.

The expression levels of the genes involved in apoptosis including p53, Bax, Bcl-2, and the ratio of Bax/Bcl-2 gene expression were determined in epicardial adipose tissues of patients with or without coronary artery diseases. Analysis of data showed that apart from the significant upregulation of p53 at both gene expression and protein level in patients with coronary artery disease as shown in Figure 1(Fig. 1), CAD patients showed a significantly higher Bax gene expression level (p < 0.001) compared to patients from control group (Figure 3(Fig. 3)). The expression of Bcl-2 gene did not differ between groups and an increasing trend was observed for Bax/Bcl-2 ratio in CAD patients; however, it did not reach statistical significance (Figure 3(Fig. 3)). 

### Concerted alterations of Sirt1, p53, Bax, and Bcl-2 expression levels in CAD

**Figure 4:**
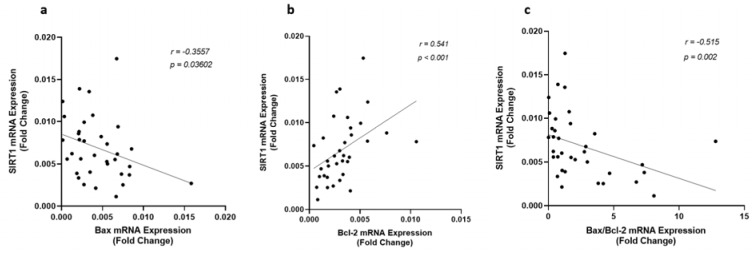
Correlation of Sirt1 gene expression level with (a) Bax, (b) Bcl-2, and (c) the ratio of Bax/Bcl-2 gene expression levels, as determined by qRT-PCR in EAT of CAD patients (n=35) compared with the control group (n=29). Spearman's correlation coefficient (r) was used to examine the relationship between the variables.

**Figure 5:**
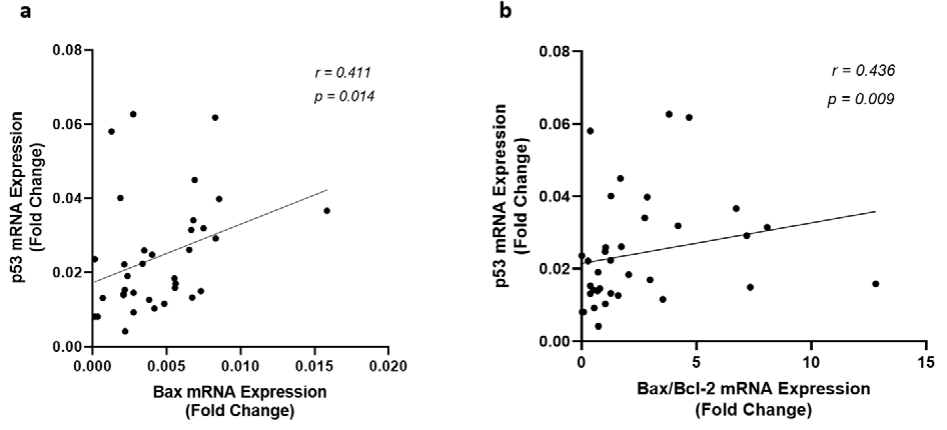
Correlation of p53 gene expression level with (a) Bax and (b) the ratio of Bax/Bcl-2 gene expression levels, as determined by qRT-PCR in EAT of CAD patients (n=35) compared with the control group (n=29). Spearman's correlation coefficient (r) was used to examine the relationship between the variables.

Since Sirt1 modulates apoptosis through regulating the expression of the genes involved in apoptosis, correlation among the expression of Sirt1, p53, Bax, Bcl-2, and the ratio of Bax/Bcl-2 were investigated in all patients. There was no significant correlation among these variables in the control group, however, the gene expression and protein levels of Sirt1 and p53 were found significantly correlated to each other (Figure 2(Fig. 2)) and with the expression of Bax and Bcl-2 in CAD patients. An inverse correlation was observed between Sirt1 and Bax gene expression levels whereas Sirt1 increased (r = 0.541, p < 0.001) by the incline in the expression of Bcl-2, as shown in Figure 4(Fig. 4). A strong negative correlation of Sirt1 and the Bax/Bcl-2 ratio of gene expression was also observed in patients with coronary artery disease. Unlike Sirt1, p53 showed positive correlations between both Bax (r = 0.411, p = 0.014) and Bax/Bcl-2 ratio (r = 0.436, p = 0.009) of gene expressions (Figure 5(Fig. 5)).

### Histological analysis

**Figure 6:**
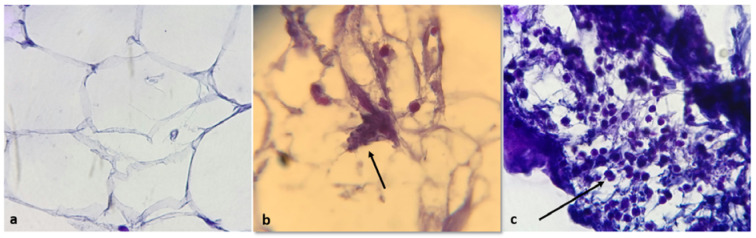
Histopathological features of EAT, as visualized by hematoxylin and eosin staining. EAT of control subjects shows absence of inflammatory cells (a) whereas EAT of CAD patients shows apoptotic bodies (b) and high infiltration of inflammatory cells mostly in periseptal areas of the fat lobules (c). Arrows indicate apoptotic bodies and inflammatory cells in b and c, respectively. Magnification × 100

Histopathological examination, as visualized by hematoxylin and eosin staining showed that epicardial adipose tissue of patients with CAD contained apoptotic bodies indicating the presence of apoptotic activity in EAT and showed some degree of inflammation, whereas EAT from control group lacked any sign of inflammation and the presence of apoptotic bodies (Figure 6(Fig. 6)).

## Discussion

Coronary artery disease (CAD) is the most common cause of death worldwide (Nguyen et al., 2019[27]). CAD generally initiates with a chronic inflammatory process, leading to the production of reactive oxygen species (ROS), and finally results in the build-up of the atherosclerotic plaque in the vessels (Regmi and Siccardi, 2020[29]). Epicardial adipose tissue (EAT) which is directly in contact with the myocardium and coronary vessels (Gaborit et al., 2017[11]; Iacobellis and Bianco, 2011[18]), mediates inflammation and plays significant roles in the pathogenesis of CAD by producing a variety of active proteins including anti- and pro-inflammatory adipokines (Iacobellis et al., 2005[19]). Furthermore, there is strong evidence that apoptosis plays an important role in the progression of atherosclerosis. In fact, the level of apoptotic cell death is dependent on the stage of atherosclerotic lesion and plaque rupture (Van Vre et al., 2012[36]). 

Various cellular signal transduction pathways, responsible for proliferation and apoptosis, are involved in the network of oncogenes and tumor suppressor genes (Suzuki et al., 2014[32]). Among them, p53 a tumor-suppressor protein with both antiproliferative and proapoptotic actions (Tabas, 2001[34]) is known to be activated in the plaque and regulates cell cycle arrest, cell senescence and apoptosis (Mercer and Bennett, 2006[25]). Since deacetylation (and deactivation) of p53 itself is regulated by Sirt1, it was hypothesized that the higher activity of p53 in epicardial adipose tissue of CAD patients might be due to the reduction in the expression of Sirt1. Thus, the present study for the first time, investigated the correlation between the expression of Sirt1 and p53 at both mRNA and protein levels and determined the expression of genes involved in EAT of patients with coronary artery disease. 

Our results showed that both mRNA and protein levels of Sirt1 were significantly lower in CAD patients while marked enhancements were observed for p53 in CAD patients compared to control subjects indicating the presence of an inverse correlation between Sirt1 and p53 expression in CAD. In addition, a significant increase in Bax gene expression in CAD group together with the strong associations of Sirt1 and p53 with the Bax/Bcl-2 ratio and the findings of histopathological examination confirmed existence of inflammation and apoptosis in epicardial adipose tissue of CAD patients. 

In p53-dependent apoptosis pathway following receiving apoptotic signal, activated p53 induces production of pro-apoptotic factors such as Bax and suppresses expression of anti-apoptotic agents like Bcl-2, and finally leads to the occurrence of cellular apoptosis. Therefore, tissues receiving apoptotic signals are expected to be associated with the higher levels of p53 and Bax proteins (Aubrey et al., 2018[4]; Elmore, 2007[9]). The contribution of higher p53 in apoptosis has been reported in a post-mortem study in patients with coronary artery disease (Blin et al., 2013[5]). Similarly, in the present study we showed that p53 gene expression and protein levels were almost doubled in patients with CAD compared to controls. This observation was somehow expected since the higher level of p53 in atherosclerosis has already been reported. 

However, here for the first time we showed that the enhancement in the expression of p53 was accompanied by reduced gene expression and protein level of Sirt1 in epicardial adipose tissue of patients with coronary artery disease. The observed inverse correlation between Sirt1 and p53 can be explained by the fact that Sirt1 is capable of modulating p53 activation (Lee and Gu, 2013[21]). p53 is ubiquitously expressed as an inactive transcription factor in all cell types throughout the body (Suzuki et al., 2014[32]) where different mechanisms including phosphorylation, dephosphorylation, acetylation, and changes in protein conformation have been proposed for regulation of its activity (Reisman et al., 2012[30]; Yamamoto et al., 2007[37]).

Sirt1, which is a member of Sirtuins family and belongs to the class III of histone deacetylase enzymes, deacetylates and inactivates p53 as substrate protein (Lee and Gu, 2013[21]). Sirt1 also deacetylates p53 C-terminal lysines and affects p53 transcriptional activity (Lee and Gu, 2013[21]). Consistent with these reports our results showed that decline in Sirt1 expression had considerable influence on p53 gene transcriptional activity and significantly increased p53 mRNA and protein levels. Similar to our findings, decreased expression levels of Sirt1 have been observed in diseases associated with oxidative stress and inflammation such as atherosclerosis (Stein and Matter, 2011[31]; Yamamoto et al., 2007[37]). Ample evidence supports the causal relationship of reduced levels of Sirt1 expressions and atherosclerosis by reporting downregulation of Sirt1 in monocytes (Breitenstein et al., 2013[6]; Chan et al., 2017[8]) and in atherosclerotic areas (Thompson et al., 2014[35]) in CAD patients and the effects of Sirt1 in preventing the formation of foam cells in endothelial cells (Stein and Matter, 2011[31]), but simultaneous determination of Sirt1 and p53 levels in epicardial adipose tissue has not been investigated so far in coronary artery diseases.

Sirt1 deacetylates and inactivates p53 and consequently inhibits its functions, particularly in p53-dependent apoptosis (Gottlieb and Oren, 1998[13]). The importance of p53 deacetylation by Sirt1 and increasing cell survival has been shown (Hori et al., 2013[16]) and the relationship between reduced Sirt1 expression, decreased its inhibitory effect on p53 expression and activity, and increased apoptosis has been reported in previous studies (Cardellini et al., 2009[7]; Peck et al., 2010[28]). In addition, it is believed that elevated levels of p53 is responsible for the evoking of antiproliferative and proapoptotic responses by a combination of transcriptional activation (e.g. Bax) or suppression (e.g. Bcl-2), and protein-protein interaction (e.g. caspases) (Elmore, 2007[9]; Tabas, 2001[34]). In line with previous reports, in the present study a higher Bax gene expression was observed in the EAT of CAD patients compared to controls but there was no difference in Bcl-2 expression between CAD patients and controls. The alteration in Bax gene expression is also consistent with previous reports indicating that pro-apoptotic protein Bax was found higher in lipid-laden macrophages in atherosclerotic lesions (Kutuk and Basaga, 2006[20]) or the Bcl-2 expression did not differ between atherosclerotic and non-atherosclerotic apoptotic cells (Kutuk and Basaga, 2006[20]). Although the Bcl-2 gene expression did not differ between groups, strong inverse correlation of Sirt1 and positive association of p53 with the Bax/Bcl-2 expression ratio was observed in this study. 

Our histopathological results also clearly showed the presence of inflammation and apoptotic bodies in epicardial adipose tissue of CAD patients. Therefore, this conclusion can be drawn that the reduction in Sirt1 resulted in the increase in p53 transcription and reduced deacetylation of p53, and increased proapoptotic activity of p53, thereby downstream gene (Bax) expression was upregulated. Lack of significant differences in Bcl-2 gene expression between groups, as observed in the present study, might be due to (i) the effects of transcription factors other than p53 on Bcl-2 expression (Kutuk and Basaga, 2006[20]; Zhou et al., 2018[39]), (ii) the possible confounding effects of other transcription factors (Yin et al., 2018[38]), (iii) oxidative stress status in EAT (Liu et al., 2016[23]) and (iv) irrelevancy of Bcl-2 expression to the presence of atherosclerosis (Kutuk and Basaga, 2006[20]).

Although the present study for the first time showed the inverse correlation of Sirt1 and p53 in epithelial adipose tissue, but taking into account the limitations present in our study, we believe that separate determination of acetylated and deacetylated forms of p53, assessment of the active forms of Bax and Bcl-2 proteins, performing a TUNEL test to assess apoptosis at the DNA level, measurement of inflammatory cytokines, and determination of oxidative status may strengthen the results. It should also be noted that because of inapplicability of collecting tissue samples, it is not usually possible to directly investigate the correlation of Sirt1 and p53 and apoptosis in cardiac myocytes.

In conclusion, the results of this study, along with findings from previous reports, confirmed the protective role of Sirt1 in atherosclerosis. It is therefore postulated that enhancement of Sirt1 expression or activity in EAT might be considered as a therapeutic strategy to impend development and progression of CAD through suppressing of the p53-dependent apoptosis in atherosclerotic area. 

## Acknowledgements

This study was based on the PhD thesis (Project Number: UMSHA-9512177682) and authors would like to thank Hamadan University of Medical Sciences for financial support.

## Funding

This research did not receive any specific grant from funding agencies in the public, commercial, or non profit sectors.

## Conflict of interest

The authors report no conflict of interest.

## Supplementary Material

Supplementary data

Supplementary material

## Figures and Tables

**Table 1:**
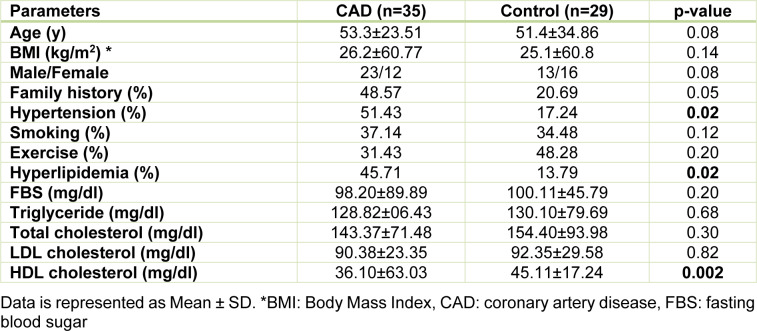
Demographic characteristics and biochemical parameters in CAD and control groups
