# Supplementary material for: Downregulation of Sirt1 is correlated to upregulation of p53 and increased apoptosis in epicardial adipose tissue of patients with coronary artery disease
Source: EXCLI J. 2020 Oct 2;19:1387–98. doi: 10.17179/excli2020-2423 (PMC7689241; doi:10.17179/excli2020-2423)
Supplement: Supplementary material [file EXCLI-19-1387-s-002.pdf]

EXCLI Journal 2020;19:1387-1398 – ISSN 1611-2156
Received: May 16, 2020, accepted: October 01, 2020, published: October 02, 2020

Supplementary material to:

DOWNREGULATION OF SIRT1 IS CORRELATED TO UPREGULATION OF p53 AND INCREASED APOPTOSIS IN EPICARDIAL ADIPOSE TISSUE OF PATIENTS WITH CORONARY ARTERY DISEASE

Mahdieh Khanahmadi[a], Babak Manafi[b], Heidar Tayebinia[a], Jamshid Karimi[a], Iraj Khodadadi[a*]

[a] Department of Clinical Biochemistry, Faculty of Medicine, Hamadan University of Medical Sciences, Hamadan, Iran
[b] Department of Surgery, Faculty of Medicine, Hamadan University of Medical Sciences, Hamadan, Iran

* **Corresponding author:** Iraj Khodadadi, Professor in Clinical Biochemistry, Department of Clinical Biochemistry, Faculty of Medicine, Hamadan University of Medical Sciences, Shahid Fahmideh Street, Hamadan-Iran. Tel: +9881 38380572; Fax: +9881 38380208. E-mail: khodadadi@umsha.ac.ir; ikhodadadi@yahoo.com

http://dx.doi.org/10.17179/excli2020-2423

This is an Open Access article distributed under the terms of the Creative Commons Attribution License (http://creativecommons.org/licenses/by/4.0/).

Supplementary Table 1: Medications used by CAD patients

Medications	Frequency N (%)
**Statin**	28 (80.00%)
**Aspirin**	29 (82.86%)
**Digoxin**	11 (31.43%)
**Calcium channel blocker**	22 (62.86%)
**Beta-blocker**	21 (60.00%)
**ACEI/ARB**	17 (48.57%)
**Diuretics**	10 (28.57%)
**Nitrates**	19 (54.29%)

All CAD patients (n = 35) were under treatment with different hypotensive and blood cholesterol lowering medications. ACEI: angiotensin converting enzyme inhibitor, N: number of patients.

Supplementary Table 2: Gene expression levels in control and patients with coronary artery disease.

Variable	mRNA expression fold change Median (IQR)		
	Control	CAD	*p*-value
**SIRT1**	0.012 (0.01)	0.006 (0.00)	**0.004**
**P53**	0.015 (0.01)	0.023 (0.02)	**0.014**
**Bax**	0.002 (0.00)	0.004 (0.00)	**<0.001**
**Bcl2**	0.002 (0.00)	0.003 (0.00)	0.462
**Bax/Bcl2**	0.971 (1.26)	1.270 (3.00)	**0.007**

The results for mRNA expression levels are shown as median (IQR). CAD: coronary artery disease, IQR: interquartile range, SIRT1: Sirtuin 1.

Supplementary Table 3: Protein expression levels in control and patients with coronary artery disease.

Variable	Protein expression fold change Median (IQR)		
	Control	CAD	*p*-value
**SIRT1**	1.912 (1.11)	0.997 (0.51)	**<0.001**
**P53**	0.684 (0.31)	1.089 (0.41)	**<0.001**

The results for protein expression levels are shown as median (IQR). CAD: coronary artery disease, IQR: interquartile range, SIRT1: Sirtuin 1.

B-actin samples from CAD patients

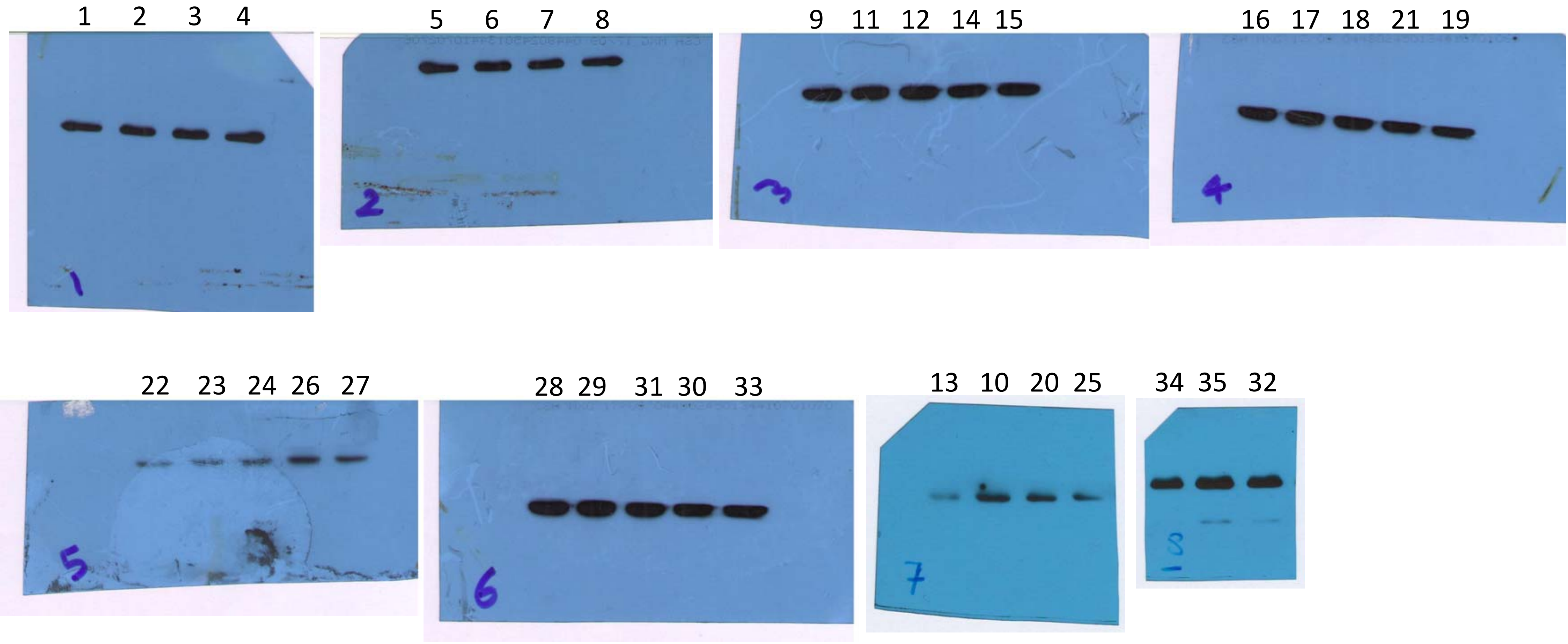

Lanes identified with **black color** represent samples from CAD patients.

B-actin samples from Control subjects

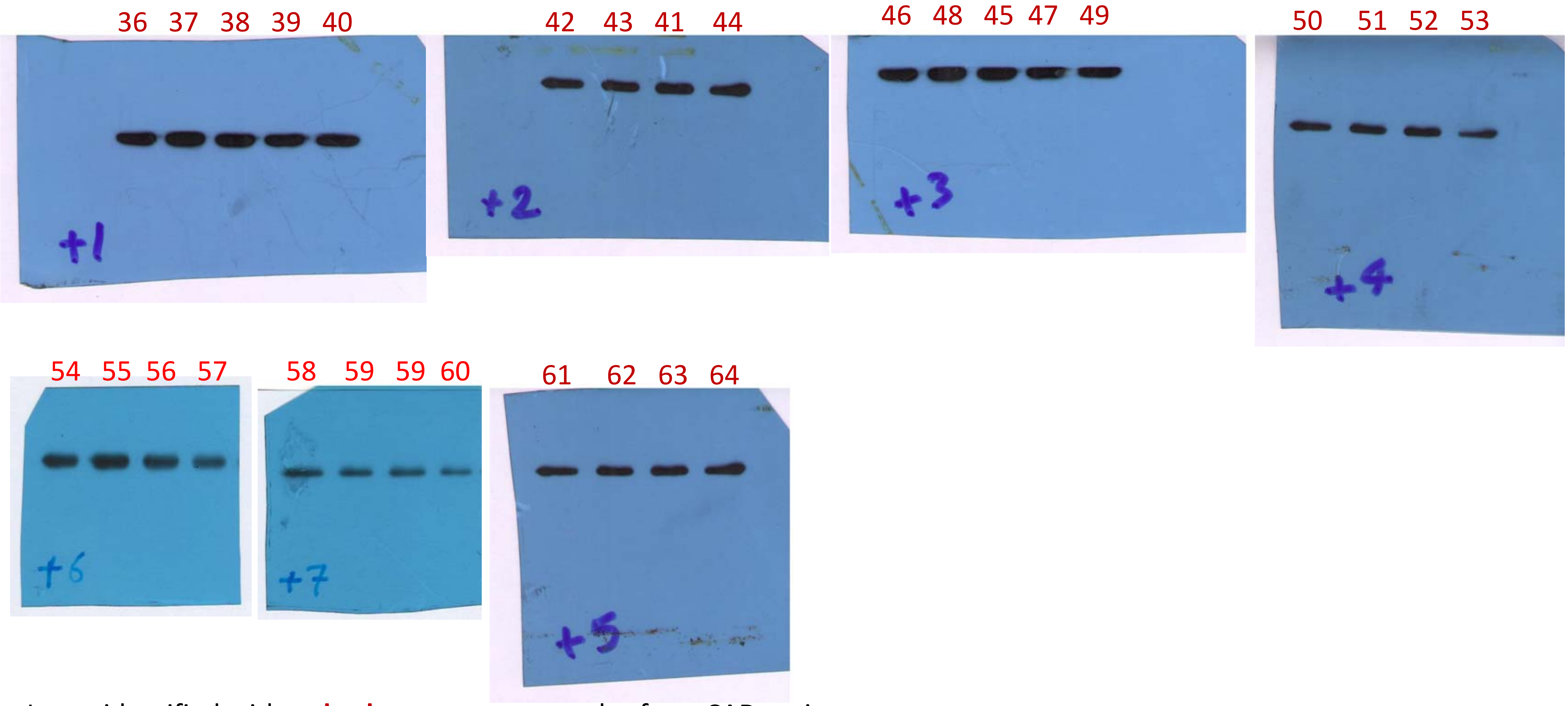

Lanes identified with **red color** represent samples from CAD patients.

P53 samples from CAD patients

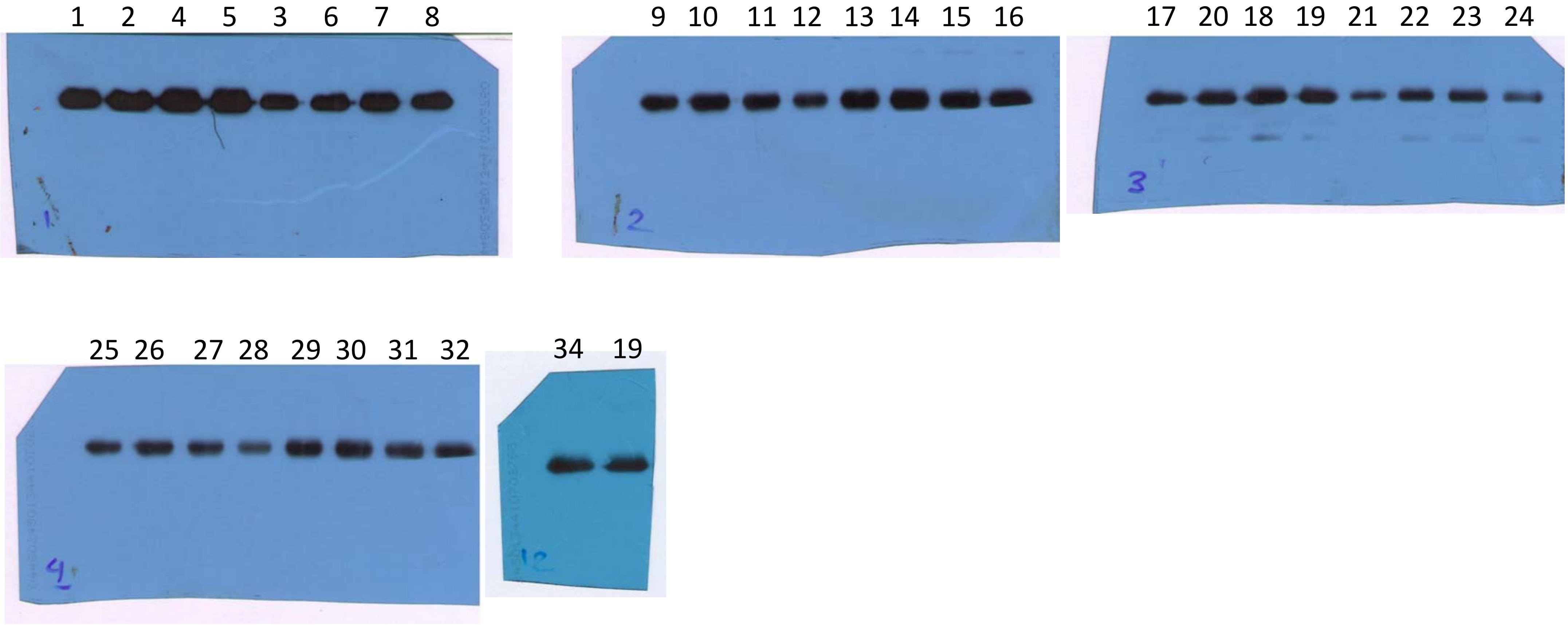

Lanes identified with **black color** represent samples from CAD patients.

P53 samples from Control subjects

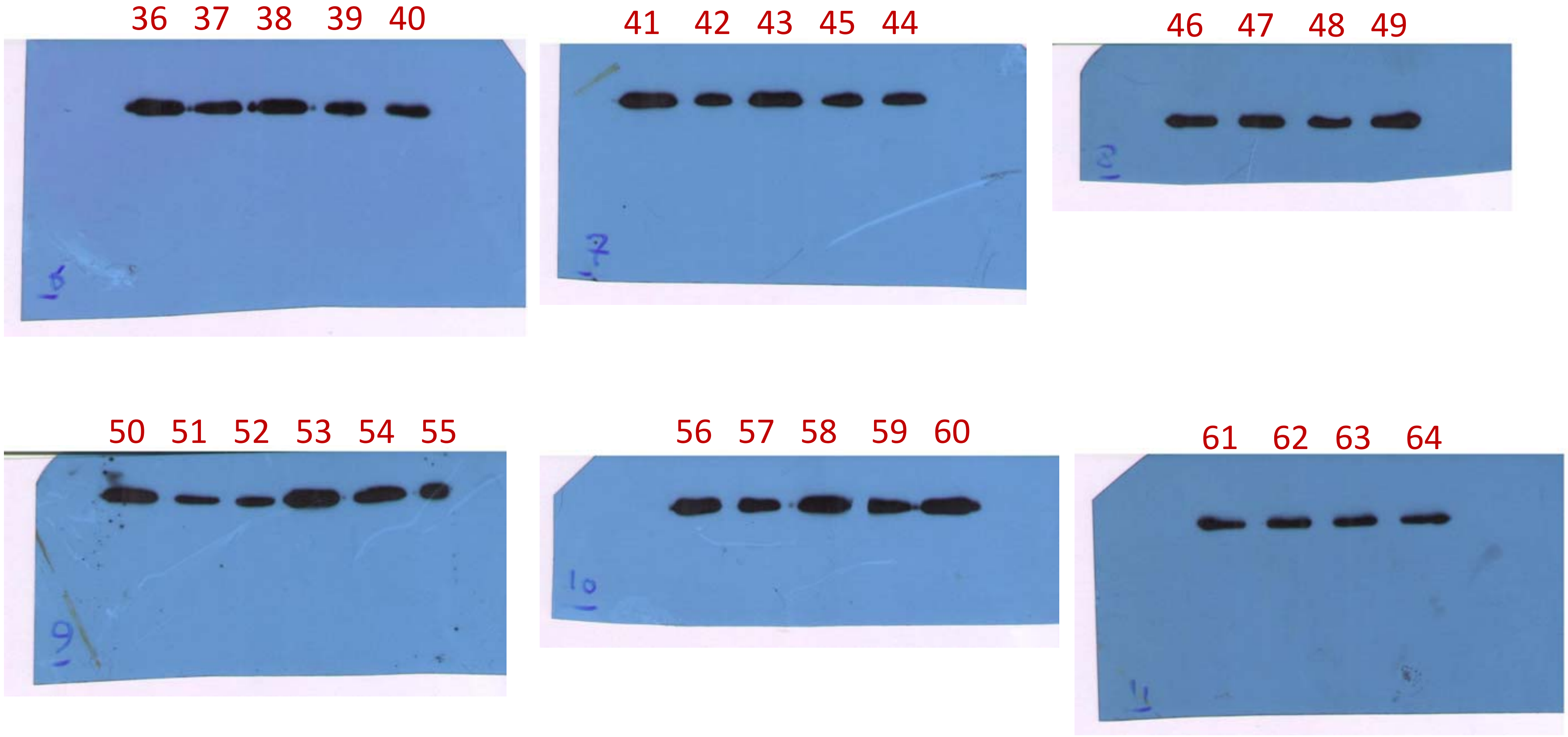

Lanes identified with **red color** represent samples from CAD patients.

P53 samples from CAD patients/Control subjects

33 35 48 47

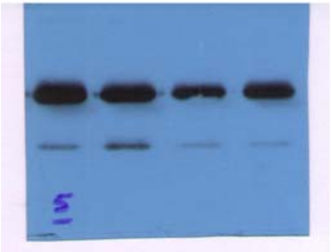

Lanes identified with **black** or **red colors** represent samples from CAD patients or control subjects, respectively

SIRT1 samples from CAD patients/Control subjects

1 2 3 4 36 37 38 39 5 6 7 8 40 41 42 43 9 10 11 12 44 45 46 47

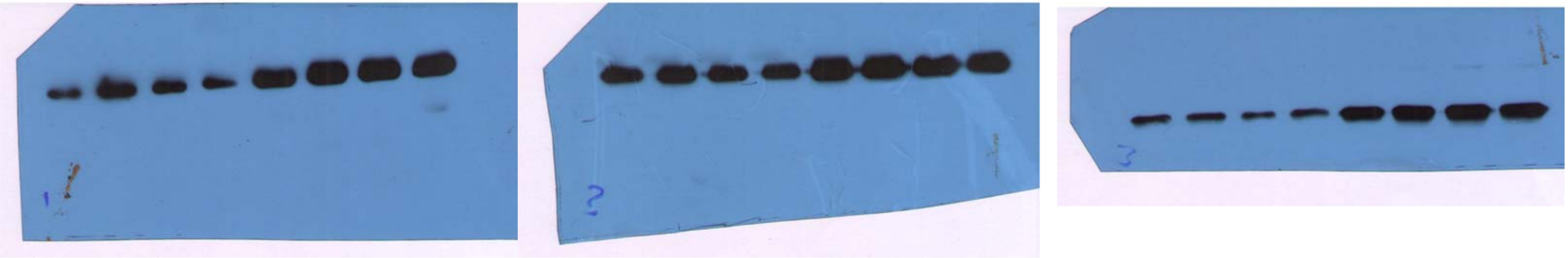

13 14 15 16 48 49 50 51 17 18 19 20 52 53 55 54 21 22 23 24 55 56 57 58

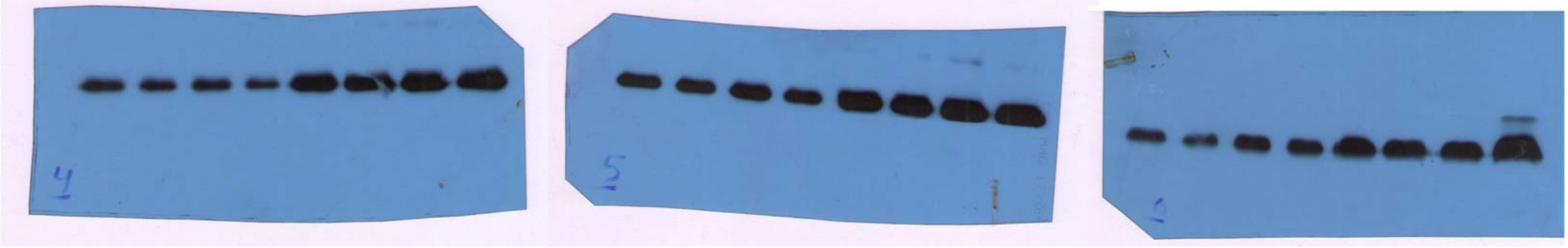

Lanes identified with **black** or **red colors** represent samples from CAD patients or control subjects, respectively

SIRT1 samples from CAD patients/Control subjects

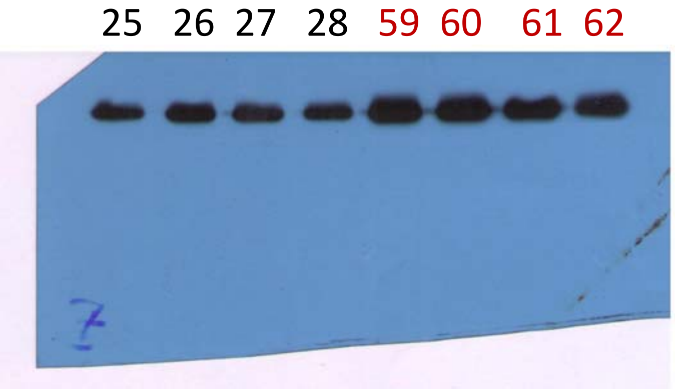

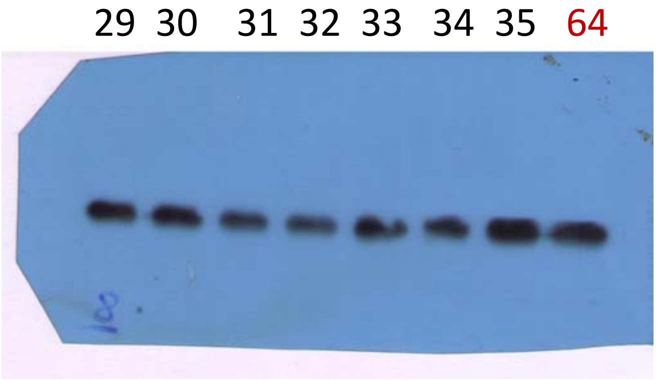

Lanes identified with **black** or **red colors** represent samples from CAD patients or control subjects, respectively
